# Influence of coracoglenoid space on scapular neck fracture stability: biomechanical study

**DOI:** 10.1186/s12891-021-04974-3

**Published:** 2022-01-04

**Authors:** Junfeng Chen, Wei Zhang, Gang Pang, Qingling Meng, Youyu Zhu, Xuefei Deng

**Affiliations:** Department of Anatomy, Anhui Medical University, Hefei, 230032 China

**Keywords:** Coracoglenoid space, Scapular neck, Fractures, Biomechanics

## Abstract

**Background:**

The anatomical variation of the coracoglenoid space has the potential to influence the stability of scapular neck fractures. This paper aimed to investigate the mechanical mechanism underlying the influence of different coracoglenoid space types on scapular neck fractures by morphometric analysis and biomechanical experiments.

**Methods:**

The morphology of 68 dried scapulae (left: 36; right: 32) was studied. Two variables, the length of the coracoglenoid distance (CGD) and the coracoglenoid notch (CGN), were measured. The distribution of CGN/CGD × 100% was used to identify the morphology of the coracoglenoid space. Each specimen was tested for failure under static axial compression loading. The average failure load, stiffness, and energy were calculated.

**Results:**

Two coracoglenoid space types were identified. The incidence of Type I (‘‘hook’’ shape) was 53%, and that of Type II (‘‘square bracket’’ shape) was 47%. The CGD and CGN were significantly higher for type I than type II (13.81 ± 0.74 mm vs. 11.50 ± 1.03 mm, *P* < 0.05; 4.74 ± 0.45 mm vs. 2.61 ± 0.45 mm, *P* < 0.05). The average maximum failure load of the two types was 1270.82 ± 318.85 N and 1529.18 ± 467.29 N, respectively (*P* = 0.011). The stiffness and energy were significantly higher for type II than type I (896.75 ± 281.14 N/mm vs. 692.91 ± 217.95 N/mm, *P* = 0.001; 2100.38 ± 649.54 N × mm vs. 1712.71 ± 626.02 N × mm, *P* = 0.015).

**Conclusions:**

There was great interindividual variation in the anatomical morphology of the coracoglenoid space. Type I (hook-like) spaces bore lower forces, were less stiff, and bore less energy, which may constitute an anatomical predisposition to scapular neck fractures.

## Background

The scapula is a complex anatomical unit, and the coracoid process is a hook-shaped bone structure that projects anterolaterally from the superior aspect of the scapular neck [1]. The coracoid process has been a popular topic in surgical management, and its morphological variation is related to some specific shoulder pathologies [2, 3]. The coracoglenoid space is an arch-shaped space that is delimited by the coracoglenoid distance (CGD) and the coracoglenoid notch (CGN). Anatomical studies have revealed large individual variation in the CGD and CGN, which could affect the bone morphology of the coracoglenoid space [2, 4]. A recent imaging study showed that the morphological variation of the coracoglenoid space was associated with the incidence of scapular neck fractures [4].

Scapular neck fractures are caused by high-energy impact transmitted by an external object or the humeral head [1, 5] and account for approximately 5–8% of all scapular fractures [6, 7]. The scapular neck is a transitional portion between the scapular body and the glenoid cavity [8], which is displaced from the root of the scapular spine and serves the functions of maintaining the normal position of the glenoid and transmitting stress [9]. Scapular neck fractures have been an object of research since the 1840s [10]. The main types include anatomical neck fracture, surgical neck fracture, and transspious neck fracture [6]. The types of these fractures are usually defined by surgeons according to the relationship between the fracture line and the passage through the coracoglenoid space [6]. Over the following decades, radiological studies demonstrated that the line of anatomical neck fractures crossed the scapular neck and passed through superiorly between the superior pole of the glenoid fossa and the base of the coracoid fossa [6, 11, 12]. However, there is anatomical variability in the coracoglenoid space, which may influence the occurrence of fractures. Detailed knowledge of the variation of this space is crucial for understanding scapular neck fractures.

Since the biomechanical mechanism by which the anatomical variation of the coracoglenoid space affects the stability of scapular neck fractures remains unclear, there is some confusion regarding the anatomical neck fracture prevalence due to the use of incorrect CT images [13] and misunderstanding of unstable anatomical scapular neck fractures due to the “floating shoulder” variation [8]. Most studies in the literature on these fractures have been case reports [6, 11], which have caused further confusion regarding the fracture types [12]. To our knowledge, there have been few studies on the biomechanical mechanism of scapular neck fractures [14]. Therefore, the purpose of this study was to evaluate the morphological parameters of the coracoglenoid space and to determine whether differences in the structure of this space affect the biomechanics of scapular neck fractures.

## Methods

### Morphological measurement of the coracoglenoid space

All procedures implemented in this study were approved by the Ethics Committee of Anhui Medical University (Hefei, China, 20,210,495). A total of 68 dried scapulae (left: 36; right: 32) were examined; the age and sex of the donors were unknown. All specimens were from adults, with none from children or elderly individuals. The specimens were donated for research and educational purposes by the Department of Anatomy of our institution. The criteria for selecting the subjects were clear and intact features.

**Fig. 1:**
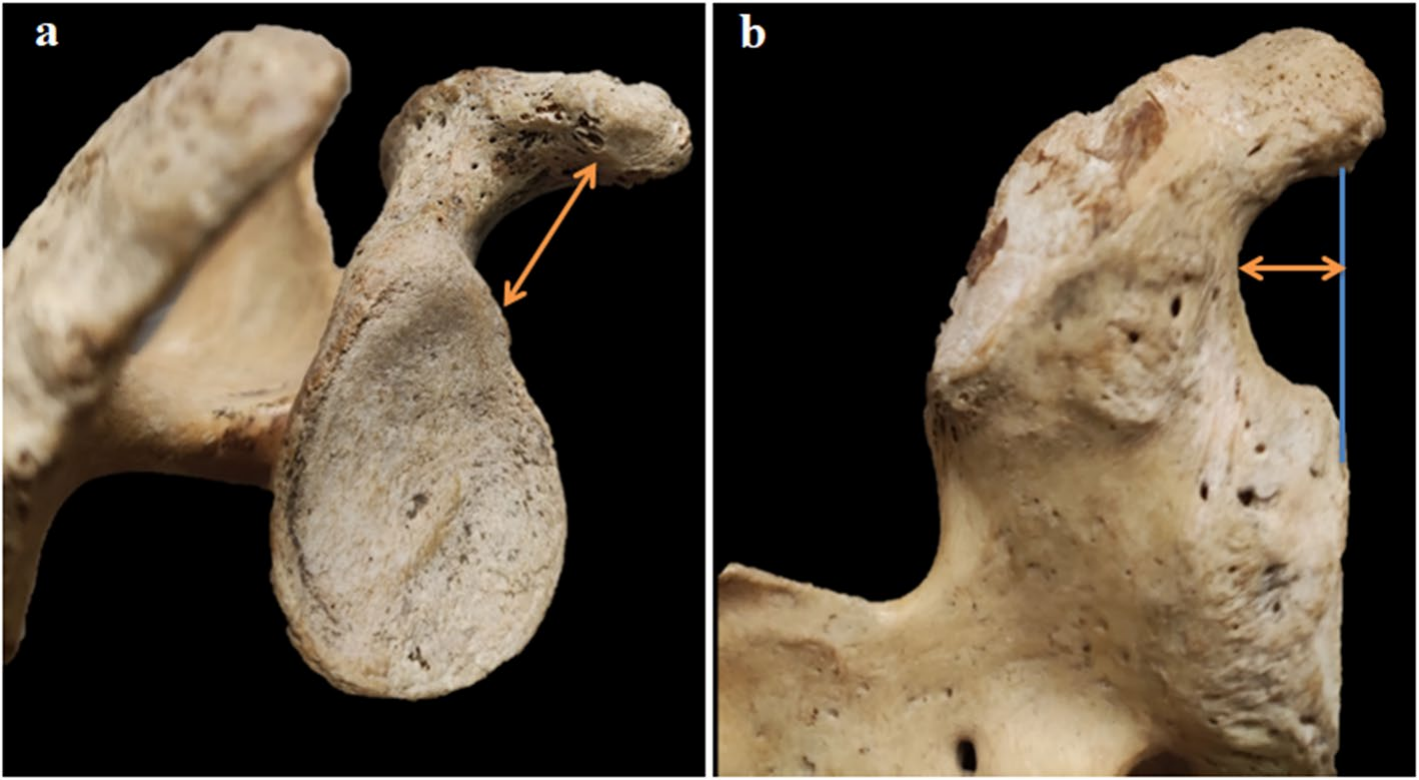
Measurement of morphological parameters of the coracoglenoid space. **a** Coracoglenoid distance (orange arrow), lateral view of the right scapula. **b** Coracoglenoid notch (orange arrow), posterolateral view of the right scapula

**Fig. 2:**
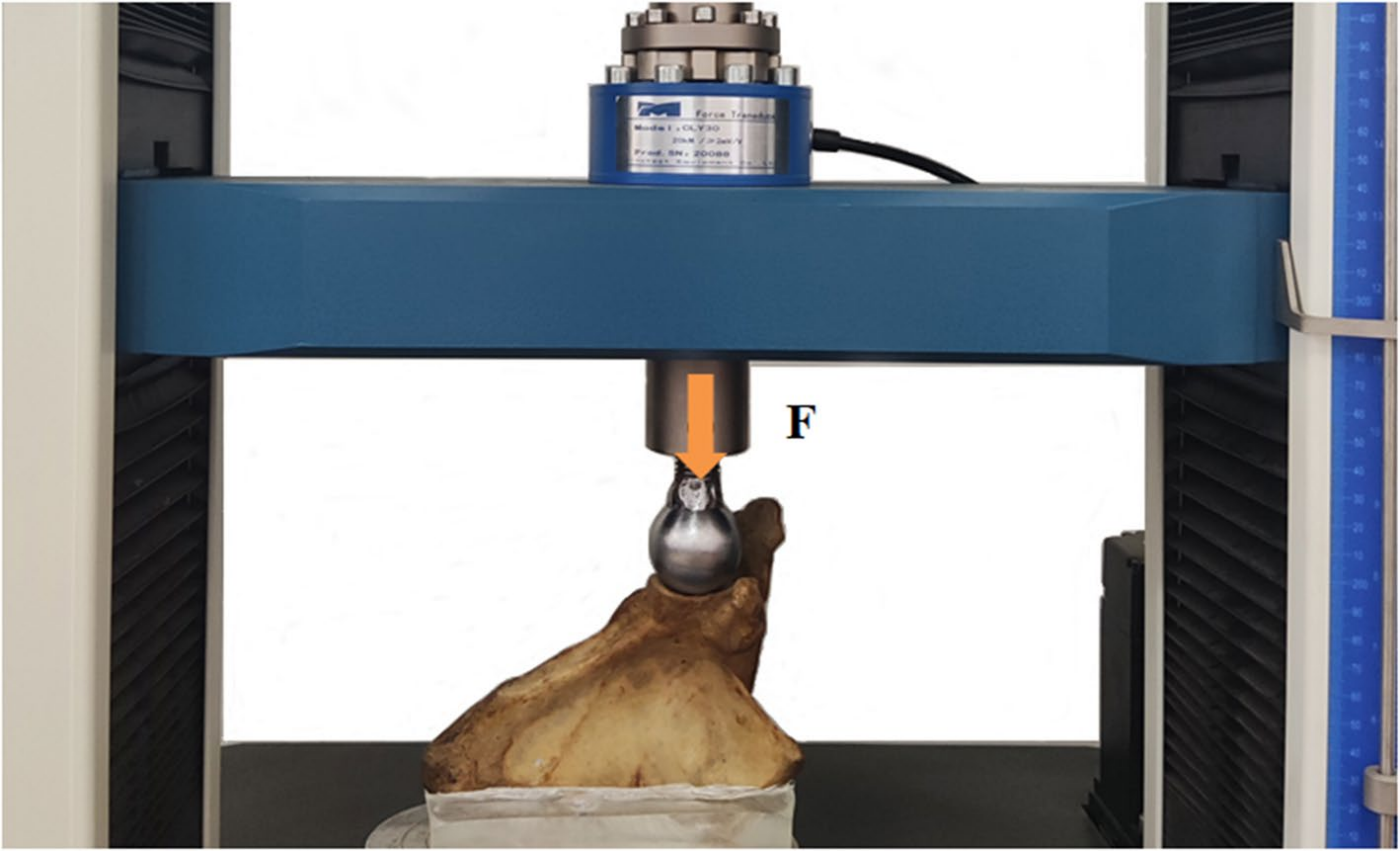
Biomechanical axial compression test setup. The test specimen was loaded in the material testing machine

The coracoglenoid space of all scapulae was analyzed. To determine the structure of this space, the following morphological parameters were used: (1) the CGD, the distance between the middle tip of the coracoid and the nearest point of the anterosuperior margin of the glenoid fossa (Fig. 1a); (2) the CGN, the distance between the upper rim of the glenoid and the coracoid base (Fig. 1b). The anatomical morphology of the coracoglenoid space was determined by the distance between the CGD and CGN. To standardize the two variables, we used the percentage of the maximal length of the CGD to express the measurement of the CGN, which corresponds to (CGN/CGD × 100%). The distribution of this ratio was used to identify the morphology of the coracoglenoid space.

Two observers used a digital caliper (the minimum scale was 0.02 mm; Qingdao, China) to measure morphological parameters of the coracoglenoid space. All specimens were measured three times by each observer, and the average was calculated. The average of the measurement results of the two observers for each specimen was taken as the final measurement.

### Specimen preparations and fixation

All selected specimens were fixed with polymethylmethacrylate (PMMA) along the medial margin of the scapula through the inferior part of the scapular spine into a custom-designed mold. The specimen was placed at the bottom of the machine, keeping the surface of the glenoid on the same plane as the compression device (Fig. 2). Based on the minimum diameter of the standard glenoid component available on the market, a custom-made 40-mm-diameter solid steel ball was used to simulate the humeral head [15] to obtain an equal load distribution on the glenoid. The ball was mounted on the beam that moved vertically downward to simulate the compressive impact of external forces or trauma on the glenoid fossa.

### Biomechanical testing

Biomechanical tests were conducted using an electronic universal tester (Model DNS-20; Sinotest Equipment Co., Ltd.; Changchun, China). Each specimen was statically loaded up to failure by the application of an increasing axial compressive load at a speed of 1 mm/min that was applied perpendicular to the face of the glenoid fossa [16]. Failure was defined as a marked decrease, with a peak inflection point of the load versus displacement curve.

**Fig. 3:**
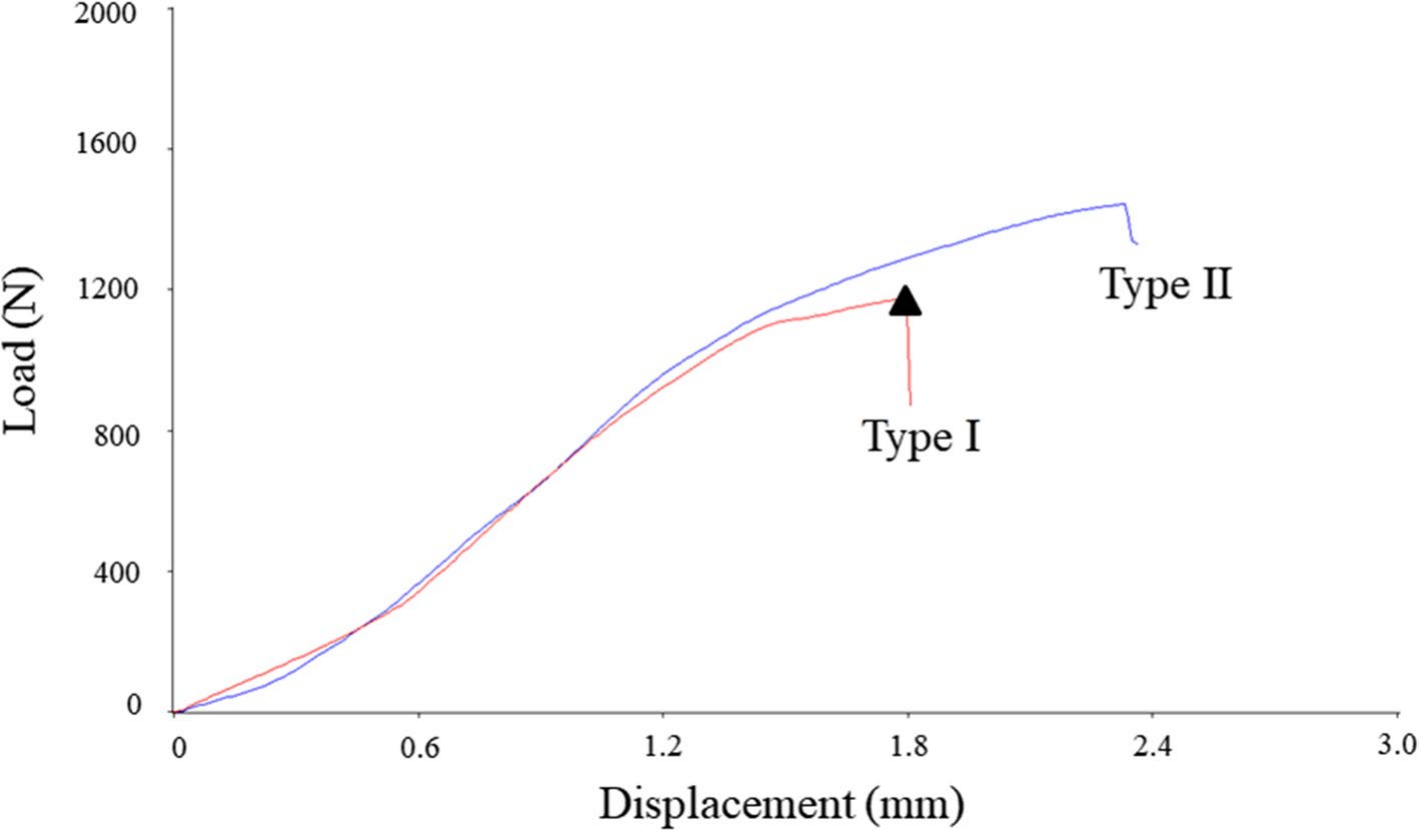
Typical load–displacement curves of the two types after increasing the load in the static axial compression test. The triangular symbol represents the maximum failure load

Load versus displacement curves were generated from the ramp and failure load data. The peak failure compression load was the highest force value attained at the point of failure of the load versus displacement curve. The failure stiffness was calculated as the slope of the linear portion of the load versus displacement curve before the yield point during failure (load divided by displacement). The failure energy was determined as the area under the load versus displacement curve (load multiplied by displacement). Each compressive force and the displacement per time were graphically shown and analyzed in all tests (Fig. 3). Measurements of the failure load, stiffness, and energy were compared. All specimens were analyzed at the point of failure to calculate the maximum biomechanical parameters.

### Statistical analysis

All data are expressed as the mean ± standard deviation. Shapiro–Wilk tests were performed to determine the normality of the data distributions. Statistical differences in the measured parameters and the biomechanical parameters of different morphological types were determined using *t*-tests. SPSS 20.0 Software (IBM Corp) was used for data analysis. Differences were defined as statistically significant at *P* < 0.05. Pearson’s correlation test was used to evaluate the correlation of two measurement variables.

## Results

### Anatomical mor*p*hology of the coracoglenoid space

**Fig. 4:**
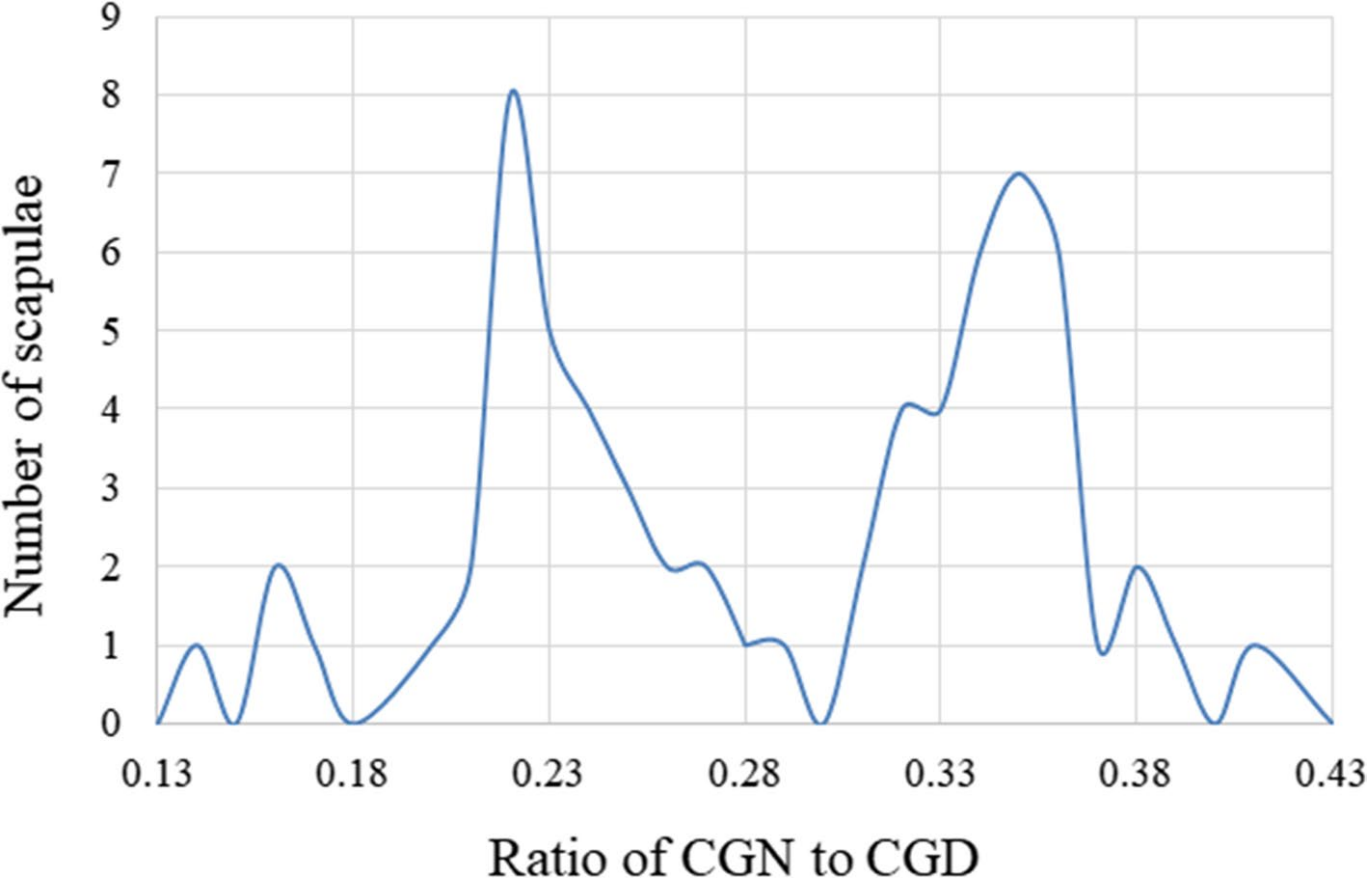
Distribution of the coracoglenoid space. CGN: coracoglenoid notch; CGD: coracoglenoid distance

The average CGD was 12.72 mm (max: 15.41 mm, min: 10.26 mm, SD: 1.46 mm). The mean CGN was 3.74 mm (max: 5.53 mm, min: 1.58 mm, SD: 1.16 mm). According to the above data, we found that the CGN corresponded to 28.79% of the CGD (SD: 6.62%). The distribution of the coracoglenoid space is shown in Fig. 4. This distribution was non-Gaussian, with two extremely different peaks located at 22% and 35%. These results show that the anatomical morphology of the coracoglenoid space is represented by two populations.

**Fig. 5:**
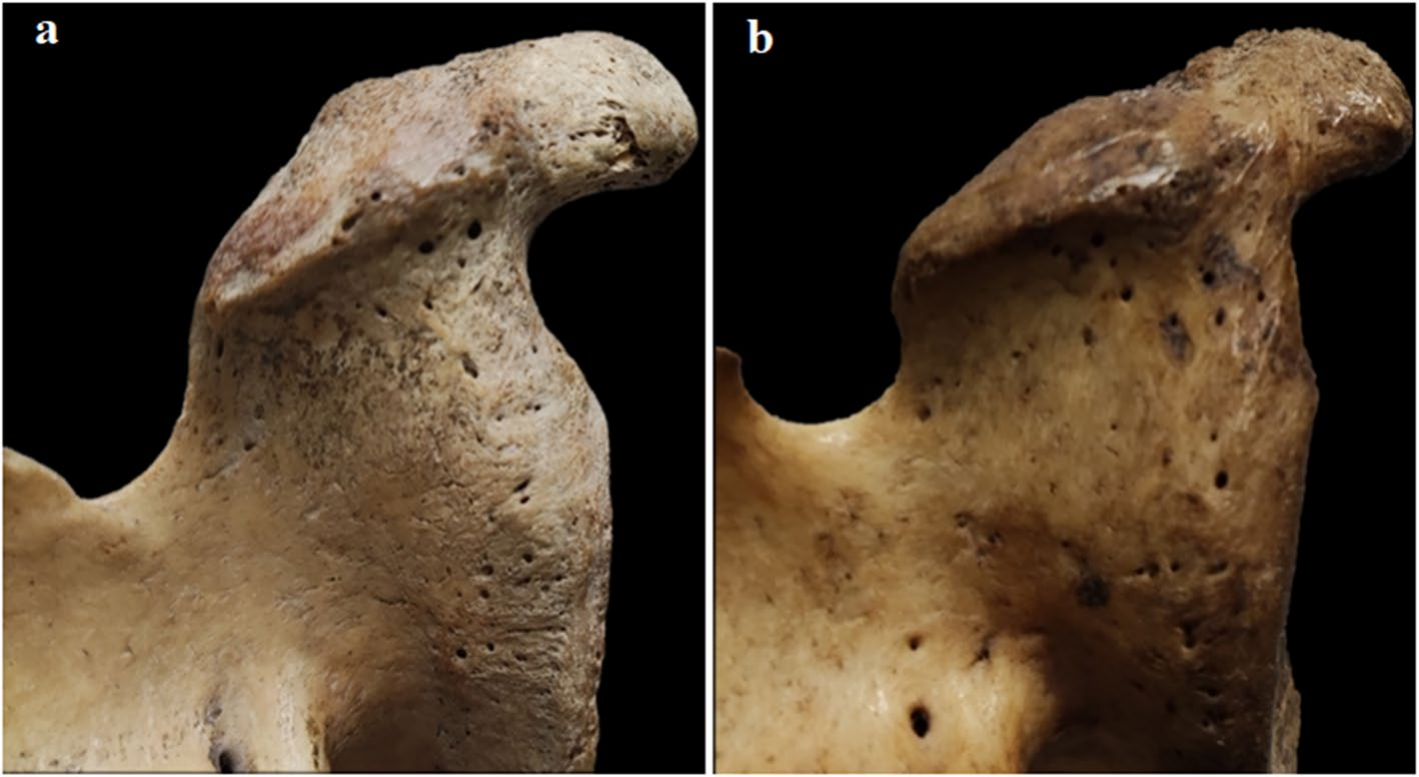
Configuration of the two coracoglenoid space type. View from the posterosuperior of the right scapula. **a** Type I (‘‘hook’’ shape). **b** Type II (‘‘square bracket’’ shape)

The coracoglenoid space within the first peak range (0.13–0.28) was classified as type I, the type I anatomical morphology exhibited a ‘‘hook’’ shape (Fig. 5a). In the second peak range (0.29–0.43), the coracoglenoid space was classified as type II, and the type II anatomical morphology exhibited a ‘‘square bracket’’ shape (Fig. 5b). The incidence rate of types I and II was 53% and 47% among all specimens. Of the 36 type I specimens examined, 17 were on the left side, and 19 were on the right side, accounting for 25.0% and 28.0%, respectively. Type II was evenly distributed on the left side and the right side (23.5% and 23.5%, respectively). There was no significant difference in the incidence of the coracoglenoid space types between the sides of the body.

### Measurement parameters of the coracoglenoid space

**Fig. 6:**
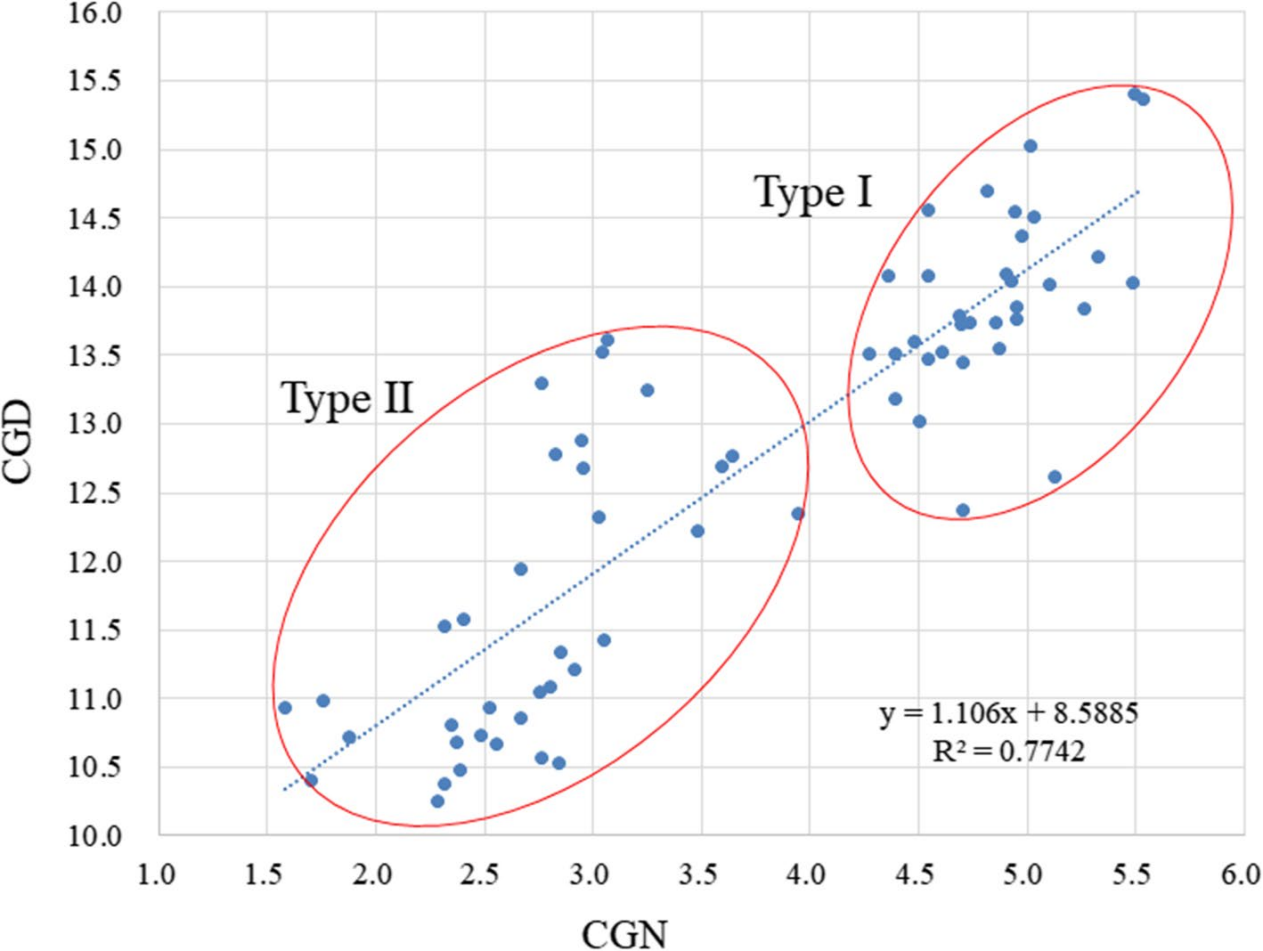
Normal distribution diagrams of two types of measurement parameters. CGN: coracoglenoid notch; CGD: coracoglenoid distance

A scatter plot (Fig. 6) was created to demonstrate the distribution of types I and II as well as the correlation between the CGN and CGD. The linear relationship between these parameters showed a significant correlation (*R*^2^ = 0.7742, *P* < 0.001). The average CGD and CGN values for type II were 11.50 ± 1.03 mm and 2.61 ± 0.45 mm, respectively, which were significantly lower than those for type I (13.81 ± 0.74 mm and 4.74 ± 0.45 mm, *P* < 0.05).

A summary of measurements regarding the CGD and CGN on different sides is shown in Table 1. For type I, the average CGD on the left and right sides was 13.88 ± 0.68 mm and 13.76 ± 0.81 mm, respectively, while for type II, the average CGD was 11.45 ± 1.02 mm and 11.54 ± 1.07 mm, respectively. The mean CGN on the left side was 4.82 ± 0.36 mm for type I and 2.65 ± 0.36 mm for type II. The mean CGN on the right side was 4.76 ± 0.52 mm for type I and 2.57 ± 0.52 mm for type II, respectively. There were no statistical differences between the left and right sides for these two types (*P* > 0.05).

**Table 1:** Comparison of the morphological parameters of the two coracoglenoid space type

Classification	Side	N (%)	CGD (mm)	CGN (mm)
	Left	17 (25.0%)	13.88±0.68	4.82±0.36
Type I	Right	19 (28.0%)	13.76±0.81	4.67±0.52
	Total	36 (53.0%)	13.81±0.74	4.74±0.45
	Left	16 (23.5%)	11.45±1.02	2.65±0.36
Type II	Right	16 (23.5%)	11.54±1.07	2.57±0.53
	Total	32 (47.0%)	11.50±1.03^a^	2.61±0.45^b^

a vs. Type I, *P* < 0.05, b vs. Type I, *P* < 0.05. There was no statistically significant different of CGD and CGN of the two types between sides of the body (*P* > 0.05)

### Biomechanical compressive parameters of the coracoglenoid space

**Fig. 7:**
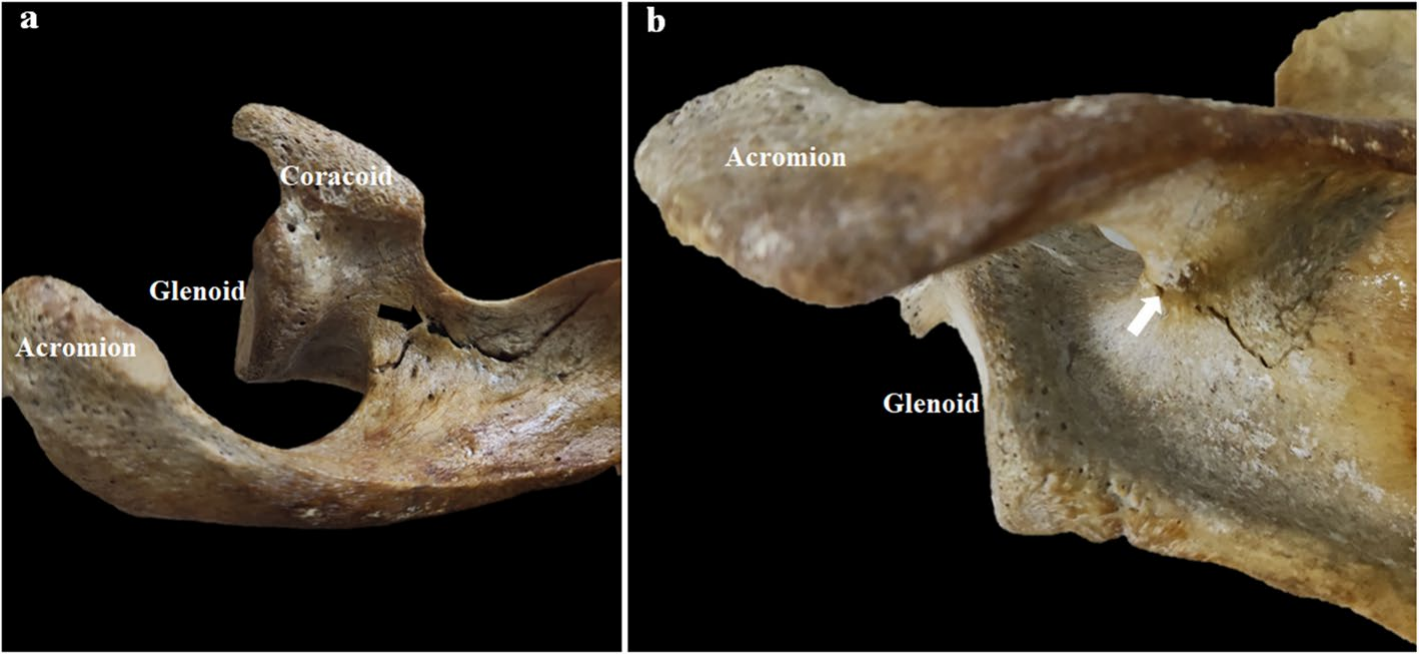
Diagram of fracture under axial load to failure. **a** anterior–superior view of acromion. **b** posterior-inferior view of acromion. The proximal fracture line started at the scapular notch area (black arrow), it ran into the spinoglenoidal notch distally (white arrow)

**Fig. 8:**
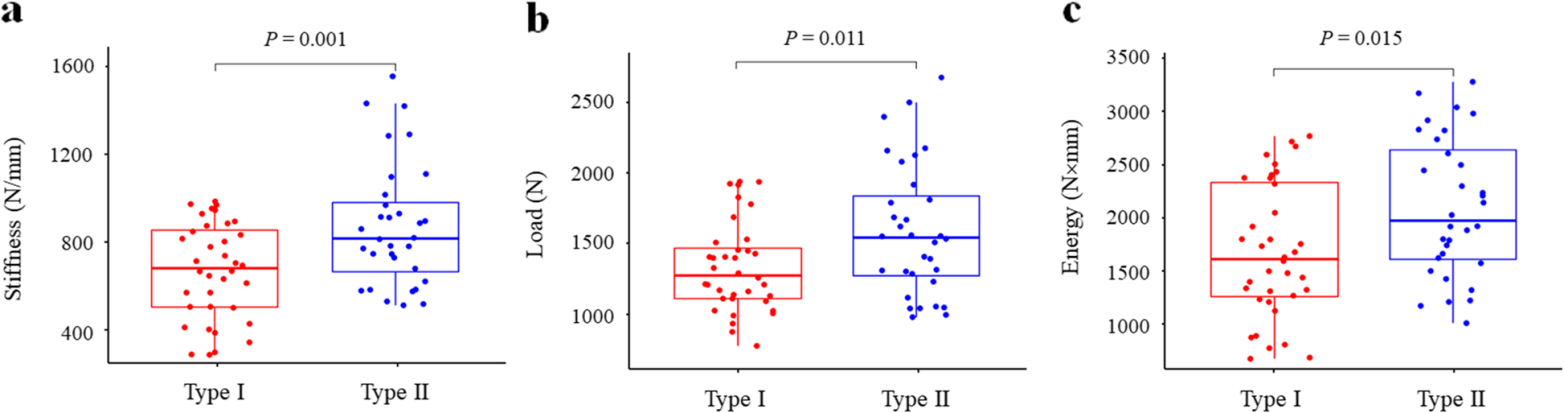
Biomechanical parameters of the two different types under axial loading. **a** Failure stiffness. **b** Failure load. **c** Failure energy

All specimens were able to withstand static axial loading up to failure, without any gross failure of PMMA during biomechanical testing. The typical fracture morphology under an axial load applied to the glenoid is presented in Fig. 7. In the destruction experiment, the proximal fracture line started at the scapular notch area. Distally, the fracture line tended to run into the spinoglenoidal notch. The mechanical properties of the two different types under compression differed significantly. The calculated results of the maximum failure load, stiffness, and energy for the two types among all specimens are illustrated in Fig. 8. The average stiffness was larger for type II (896.75 ± 281.14 N/mm) than type I (692.91 ± 217.95 N/mm) (*P* = 0.001). The mean load and energy to failure were significantly higher for type II than type I (1529.18 ± 467.29 N vs. 1270.82 ± 318.85 N, *P* = 0.011; 2100.38 ± 649.54 N × mm vs. 1712.71 ± 626.02 N × mm, *P* = 0.015).

## Discussion

Fractures caused by shoulder trauma are one of the most common conditions encountered in orthopedic surgery. More than half of extra-articular fractures involve the scapular neck [17]. The proximal part of the fracture line of scapular neck fractures reaches the space delimited by the upper border of the glenoid fossa and the base of the coracoid process (i.e., the coracoglenoid space) [12]. The shape and size of the coracoglenoid space vary greatly among individuals. Therefore, anatomical morphometric and biomechanical studies of these structures may provide information about the etiology of scapular neck fractures.

In this study, the average CGN was 3.74 ± 1.16 mm, which is consistent with the anatomical research results reported by Strnad et al. [4]. Ott et al., a German scholar [18], reported an average CGN size of 6.5 ± 2.1 mm, which is larger than that in the present study. The mean CGD in our study was 12.72 ± 1.46 mm. This is contrary to the mean CGD of 20.0 ± 4.0 mm reported by Alobaidy et al. [2]. This inconsistency may be due to differences in ethnicity. The specimens in our study were from Asians. Previous studies have shown that the dimension of the coracoid process is significantly smaller in Asians than in Europeans [19]. An interesting finding of this study was that there was a significant linear correlation between the CGN and CGD. This finding further shows that anatomical variation of the coracoglenoid space is a common occurrence.

Through this study, we found two coracoglenoid space populations. Although it was very simple to determine the anatomical variation based on the CGD and CGN, this approach served our purpose of assessing morphological differences in the coracoglenoid space. In type I, the average CGD and CGN was 13.81 ± 0.74 mm and 4.74 ± 0.45 mm, respectively, and these values were significantly higher than those in type II (11.50 ± 1.03 mm and 2.61 ± 0.45 mm, respectively). The type I morphology shows a prominent superior pole of the glenoid and a large depression at the base of the coracoid, forming a marked hook-like coracoglenoid space. In contrast, the type II morphology appears similar to a square bracket. The contour of type I reflects a more complex morphology than type II. This result may be explained by the fact that the superior pole of the glenoid serves as the attachment point for the long head biceps tendon. During prolonged shoulder movement, the stress generated by repeated muscle fiber contraction can stimulate bone growth [4]. Consequently, the arch-like structure delimited by the base of the coracoid and the anterosuperior part of the glenoid fossa deepens.

Surgical cases of scapular neck fractures often present challenging fracture line alignment due to the complex anatomical patterns involved and the high-energy injury mechanism. Miller and Ada [8] described a new type of scapular neck fracture, i.e., type IIC, consisting of fracture of the neck inferior to the scapular spine, and controversy regarding the classification of scapular neck fractures remains. After incorporating type IIC into the classification of scapular neck fractures, Goss [12] described this type as a ‘‘fracture of the neck inferior to scapula spine’’. Jaeger et al. [20] designated an anatomic neck fracture (denoted as F0), which was defined as “a fracture of the articular segment, not through the glenoid, but resulting in the fossa being detached from any part of the scapula body’’. The challenge lies in the uncertainty regarding the possible mechanism of scapular neck fractures, which complicates the clinical diagnosis. Therefore, understanding biomechanical patterns of these fractures is of great importance in determining the fracture types.

To the best of our knowledge, no biomechanical compression tests of different coracoglenoid space types have been performed. In the biomechanical failure test, the stiffness was 896.75 ± 281.14 N/mm and 692.91 ± 217.95 N/mm for types II and I, respectively. According to the calculation results of the load versus displacement curve, the failure load and energy were significantly lower for type I than type II. From the biomechanical point of view, stiffness is defined as the resistance of a structure to deformation. The greater the stiffness is, the smaller the deformation [21]. When the applied load gradually increases to the failure load, the deformation and energy of bone reach the maximum, which will eventually lead to fracture [22].

In our study, the proximal fracture line started at the scapular notch area, and the distal fracture line tended to run into the spinoglenoidal notch, which is similar to the typical scapular neck fracture reported by Bartoníček et al. [6]. This is also consistent with the findings of a study by Strnad et al. [4], who used three-dimensional CT reconstruction to observe the relationship between the morphology of the upper rim of glenoid and coracoid base and the fracture line in scapular neck fracture patients. The overall stability of the scapula under axial loading is maintained by the lateral pillar, the area of spine, and the superior border of scapula [23]. Daalder et al. [24] showed that the attachment region of coracoid base has the lowest bone density compared with the lateral border and spine, which might explain the preferred fracture location in our biomechanical compression test. Thus, we speculated type I variation serves as an anatomical factor of predisposition to scapular neck fractures.

There are several limitations to our study. First, the number of specimens (68) was relatively small, but all of them were scapular specimens from human cadavers. As such, we could better capture the natural variation in the shape and mechanical properties of human bone. Second, the biomechanical setup in our study could not be used to calculate the power. A further drawback of this study is that we were unable to assess the effects of muscle forces in vivo because violent voluntary muscle contraction can be a reason for fracture [25]. In future work, we will focus on the influence of muscle forces acting on the shoulder in the context of these fractures.

## Conclusion

In this study, we focused on the coracoglenoid space to determine its variability. We found two morphological types of the coracoglenoid space: a hook-shaped type and a square bracket-shaped type, confirming the existence of interindividual variations. Type I (hook-like) space bore lower forces, were less stiff, and bore less energy, which may constitute an anatomical predisposition to scapular neck fractures.

## Data Availability

The data presented in this study are available on request from the corresponding author.

## Abbreviations

CGD: Coracoglenoid distance
CGN: Coracoglenoid notch
PMMA: Polymethylmethacrylate
